# Predicting resistance as indicator for need to switch from first-line antiretroviral therapy among patients with elevated viral loads: development of a risk score algorithm

**DOI:** 10.1186/s12879-016-1611-2

**Published:** 2016-06-13

**Authors:** Sarah E. Rutstein, Mina C. Hosseinipour, Morris Weinberger, Stephanie B. Wheeler, Andrea K. Biddle, Carole L. Wallis, Pachamuthu Balakrishnan, John W. Mellors, Mariza Morgado, Shanmugam Saravanan, Srikanth Tripathy, Saran Vardhanabhuti, Joseph J. Eron, William C. Miller

**Affiliations:** Department of Health Policy and Management, University of North Carolina at Chapel Hill, Chapel Hill, NC USA; Division of Infectious Diseases, University of North Carolina at Chapel Hill, Chapel Hill, NC USA; UNC Project, Lilongwe, Malawi; BARC-SA and Lancet Laboratories, Johannesburg, South Africa; YRG Centre for AIDS Research and Education (YRG CARE), Voluntary Health Services, Taramani, Chennai, 600113 India; Division of Infectious Diseases, University of Pittsburgh, Pittsburgh, PA USA; Department of STD, AIDS, and Viral Hepatitis, Brazilian National STD and AIDS Program, Rio de Janeiro, Brazil; YRG Centre for AIDS Research and Education, Chennai, India; Institute for Leprosy and Other Mycobacterial Diseases, Tajganj, Agra, India; Harvard T.H. Chan School of Public Health, Boston, MA USA; Department of Epidemiology, University of North Carolina at Chapel Hill, Chapel Hill, NC USA

**Keywords:** HIV, Resistance, Viral load monitoring, Prediction models, Resource-limited setting

## Abstract

**Background:**

In resource-limited settings, where resistance testing is unavailable, confirmatory testing for patients with high viral loads (VL) delays antiretroviral therapy (ART) switches for persons with resistance. We developed a risk score algorithm to predict need for ART change by identifying resistance among persons with persistently elevated VL.

**Methods:**

We analyzed data from a Phase IV open-label trial. Using logistic regression, we identified demographic and clinical characteristics predictive of need for ART change among participants with VLs ≥1000 copies/ml, and assigned model-derived scores to predictors. We designed three models, including only variables accessible in resource-limited settings.

**Results:**

Among 290 participants with at least one VL ≥1000 copies/ml, 51 % (148/290) resuppressed and did not have resistance testing; among those who did not resuppress and had resistance testing, 47 % (67/142) did not have resistance and 53 % (75/142) had resistance (ART change needed for 25.9 % (75/290)). Need for ART change was directly associated with higher baseline VL and higher VL at time of elevated measure, and inversely associated with treatment duration. Other predictors included body mass index and adherence. Area under receiver operating characteristic curves ranged from 0.794 to 0.817. At a risk score ≥9, sensitivity was 14.7–28.0 % and specificity was 96.7–98.6 %.

**Conclusions:**

Our model performed reasonably well and may be a tool to quickly transition persons in need of ART change to more effective regimens when resistance testing is unavailable. Use of this algorithm may result in public health benefits and health system savings through reduced transmissions of resistant virus and costs on laboratory investigations.

**Electronic supplementary material:**

The online version of this article (doi:10.1186/s12879-016-1611-2) contains supplementary material, which is available to authorized users.

## Background

The World Health Organization (WHO) recommends viral load (VL) as the preferred method for monitoring antiretroviral therapy (ART) and diagnosing viral failure in HIV-infected patients [1]. An elevated VL is an important gauge of treatment effectiveness, indicating poor adherence and/or drug resistance [2–5]. Failing to switch persons with drug resistance to second-line therapy in a timely manner increases morbidity and mortality, likelihood of second-line treatment failure, and transmission of resistant virus [2, 6–15]. Drug resistance testing is rarely available in resource-limited settings, where the majority of persons accessing ART reside [16]. Distinguishing persons with modifiable poor adherence without resistance mutations from persons with drug resistance (for whom improved adherence will not result in viral resuppression) is critical to reduce the spread of resistance and improve effectiveness of second-line therapies.

**Fig. 1:**
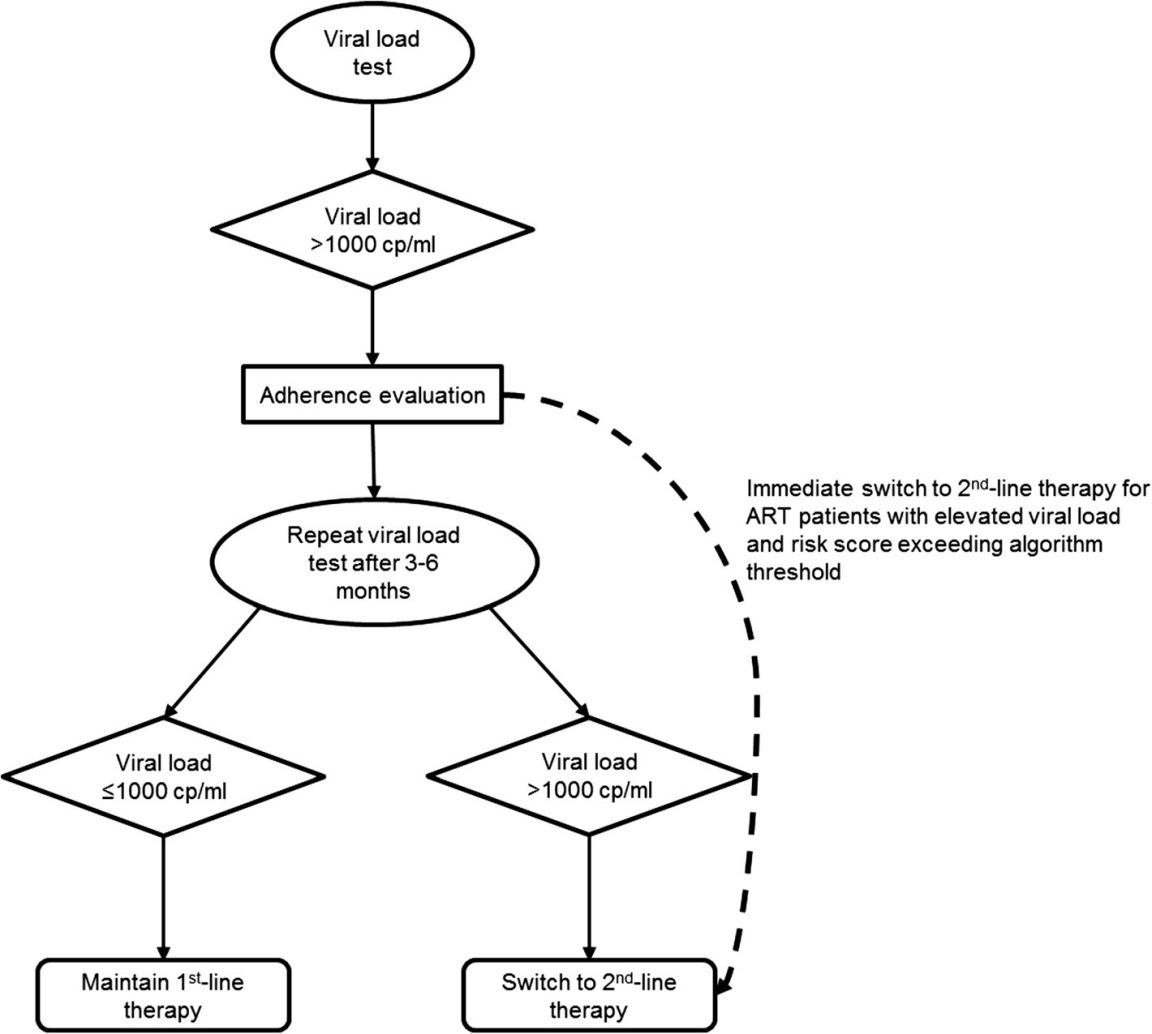
World Health Organization viral load testing strategy for treatment failure [1]. Persons eligible for viral load testing may be tested using plasma-based assays or dried blood spots. For plasma assays, a viral load >1000 copies/ml prompts an evaluation of adherence to antiretroviral therapy and targeted adherence counseling if deficiencies in adherence are observed. The viral load test is repeated 3 to 6 months later (confirmatory test). Patient management is dictated by results of this second test – patients with confirmed elevated (>1000 copies/ml) viral loads are switched to second-line therapy. The dashed arrow represents implementation of the risk score algorithm. Persons with a risk score exceeding the predefined algorithm threshold would be switched immediately to second-line therapy

Current VL monitoring algorithms require confirmatory testing for elevated initial tests (Fig. 1) [1]. This two-step process presents an opportunity for counseling that may improve adherence leading to virological resuppression [17]. However, for persons with resistant viruses, requiring a second test unnecessarily postpones the treatment switch. The delays introduced with confirmatory testing are especially relevant in resource-limited settings: programmatic and patient-related obstacles may substantially increase the interval between first and confirmatory testing. Among persons with confirmed virological failure in South Africa, switch to second-line therapy took >5 months after confirmatory VL [18, 19].

In sub-Saharan Africa, >25 % of ART persons may not achieve viral suppression by 12 months [20], and rates of virological failure may be as high as 14 % at five years [19]. With nearly ten million persons receiving ART in low- and middle-income countries [21], eliminating confirmatory testing for even a fraction of ART-resistant persons will produce substantial cost savings. Furthermore, early identification of treatment failure may avoid significant morbidity and mortality for patients who otherwise remain on inappropriate therapy.

Distinguishing persons with elevated viremia with without drug resistance mutations is challenging in resource-limited settings where resistance testing is unavailable. A simple risk score algorithm may help providers identify patients with probable ART resistance who could be switched to second-line therapy immediately without confirmatory testing. Using patient demographics, clinical, and laboratory-based predictors that would be readily available in most clinical settings, we developed a risk score algorithm to predict the need for ART change by identifying resistance among patients with persistently elevated VL. Patients exceeding a pre-specified risk score threshold could be switched immediately; patients below this threshold would have confirmatory VL testing prior to treatment switch decisions.

## Methods

### Study setting and population

Eligible participants in the Prospective Evaluation of Antiretrovirals in Resource-Limited Settings (PEARLS) trial (Adult AIDS Clinical Trials Group (ACTG) A5175, NCT00084136) were studied. PEARLS was an open-label, Phase IV, randomized clinical trial that investigated efficacy and safety of once- vs. twice-daily regimen dosing. Details of the PEARLS study population and design have been described elsewhere [22]. In brief, A5175 enrolled 1571 HIV-infected participants ≥18 years old from nine countries, over-sampling participants from resource-limited settings. Participants were excluded from PEARLS if they: had a CD4 cell count >300 cells/mm^3^, previous exposure to ART (exception for women who received ART for prevention of mother-to-child transmission), were pregnant, or were acutely ill and/or clinically unstable. PEARLS was approved by institutional review boards and ethics committees at participating institutions.

This study is a post-hoc analysis of a subset of de-identified data restricted to participants initiated on non-nucleoside reverse transcriptase inhibitor (NNRTI)-based regimens (lamivudine/zidovudine/efavirenz or emtricitabine/tenofovir/efavirenz) who had at least one VL ≥1000 copies/ml at any point after week 16 of enrollment. This population was distinct from the A5175 definition of failure which required two consecutive measurements of plasma HIV-1 RNA ≥1000copies/ml after 16-weeks or disease progression. Primary analyses included participants from all study sites; a sensitivity analysis restricted the population to participants enrolled from resource-limited settings. This analysis was approved by the University of North Carolina, School of Medicine Institutional Review Board.

### Data collection

Per A5175 study protocol, participants received a targeted physical exam, adherence interview, serum chemistries, CD4 lymphocyte count, and plasma HIV RNA (Roche Amplicor Monitor assay [v1.5]) at least every eight weeks. Any treatment modification (participant, provider, or protocol-mandated) was assessed at each visit. Diagnosis criteria were collected using a standardized case report form.

Resistance tests were done retrospectively at four regional laboratories participating in the National Institute of Allergy and Infectious Diseases Division of AIDS Virology Quality Assurance program, coordinated by the HIV Prevention Trials Network Laboratory Center, using ViroSeq HIV-1 Genotyping Assay (Celera Diagnostics, Alameda, California) on stored specimens [23]. Resistance testing was done for participants meeting study-specific virological failure criteria (defined below) or who had disease progression (new or recurrent AIDS-defining opportunistic infection or malignancy) ≥12 weeks after randomization.

### Measures

The outcome (need for ART change after first VL ≥1000 copies/ml) was assessed using the following algorithm: resuppression (<1000 copies/ml) – no ART change needed; no resuppression and no resistance – no ART change needed; and no resuppression and resistance – ART change needed. Participants with NRTI or NNRTI resistance mutations, defined by 2014 International AIDS Society guidelines (excluding mutation 101P), were classified as resistant [24]. Resistance to protease inhibitors (PI) were not included; this class of drugs is reserved for second-line therapy and thus, if observed in the absence of NRTI/NNRTI resistance, would not be an indication for treatment change from first-line regimen. We did not distinguish between baseline and acquired resistance. Resistance testing was not done on participants who had a VL ≥1000 copies/ml and resuppressed at their subsequent study visit. Any participant who resuppressed was classified as not needing ART change. Participants who had two sequential study visits with VL ≥1000 copies/ml, but who did not have a resistance test, were excluded.

Potential predictors of needing ART change included demographics, clinical diagnoses prior to treatment initiation, immunological markers (CD4 cell count), self-reported and provider-assessed ART adherence, and therapy duration (based on the number of days between ART initiation and a participant’s first VL ≥1000 copies/ml). Per WHO and other country ART guidelines, the six-month visit is frequently identified as the first point that a participant is eligible for VL monitoring [1, 25, 26]. A six-month visit was defined as any time point ≥16-week visit and ≤ 212 days after ART initiation; this time frame includes an acceptable 30-day extension of the six-month window period. The 12-month visit was similarly classified as any time after the six-month window up to and including 30 days after 12 months on ART (395 days).

### Statistical analyses

All analyses were conducted using Stata statistical software (Version 13.0; Stata Corporation, College Station, TX).

We constructed three multivariable models to predict need for ART change that reflected variations in availability of CD4 and VLs at time of ART initiation. Although many countries have scaled up access to CD4 testing to determine ART eligibility, the roll-out of Option B+, in which HIV-infected pregnant women are initiated on lifelong ART regardless of CD4, could mean that many persons will not have a CD4 cell count at treatment initiation [1]. In light of these policies and the capacity constraints in resource-limited settings, models were constructed to reflect three scenarios:

- **Model 1** assumed that VL and CD4 at ART initiation were available, so both were included as eligible predictors.
- **Model 2** assumed that treatment initiation CD4 was available but that treatment initiation VL was not and thus excluded as an eligible predictor.
- **Model 3** assumed that neither treatment initiation VL nor CD4 were available; thus neither was included as eligible predictors.

To evaluate the association between predictors and need for ART change, we calculated unadjusted prevalence odds ratios (OR) and 95 % confidence intervals (CI) for each potential predictor in each model [27].

The full models contained all variables with bivariate *p*-values <0.5; this high threshold was chosen to ensure that available important predictors were considered [28]. Variables with low frequency, extreme collinearity, or insufficient detail to permit clinical implementation were excluded, regardless of *p*-value. We tested four categorizations of time on treatment and selected the category with the lowest Akaike’s information criteria (AIC) value for our reference models [29].

We developed the predictive models using multiple logistic regression with backward elimination [27]. Beginning with the variable with the largest p-value, we removed variables one at a time until ≤5 remained (regardless of *p*-value). The five-variable limit was selected to facilitate eventual implementation of risk scores in resource-limited clinical settings [30, 31]. We assessed the equality of the area under the receiver operating characteristic curves (AUROC) between each model (chi-squared test) [32]. AUROC measures the risk score’s discriminatory power –where 1.0 indicates a perfect test (i.e., 100 % sensitivity and 100 % specificity) [33]. Likelihood ratio (LR) comparing successive models were evaluated to confirm that variable removal did not adversely affect the model’s predictive capacity. We also compared LR-test statistics from each reduced model to the full model.

We used the three predictive models to develop the associated risk scores by assigning each variable in the final models a predictor score equal to two times the beta coefficient rounded to the nearest integer. We doubled the coefficient to retain inherent discrimination between betas, while keeping the absolute numbers manageable. Patients with a high VL (≥1000 copies/ml) and a risk score equal to or greater than a pre-specified cutoff are classified as likely needing to switch to second-line ART without a confirmatory VL test. For each model, we assessed sensitivity, specificity, and associated risk scores at cutoffs selected based on clinically-acceptable model-performance criteria [34, 35]. Given the undesirable consequences of prematurely switching persons to second-line therapy, we maintained a high specificity threshold (>95.0 %) for all models to minimize false positives. We also calculated the number of patients in a hypothetical cohort of 10,000 ART patients who would be switched without confirmatory testing at each cutoff. We internally validated the model and risk score performance using 1000 bootstrap samples with replacement [27, 36]. Bootstrapping is a process in which each iteration resamples a random cohort of data points from within our full dataset and assess validity of our calculated estimates using this “new” sample. Bootstrapping is preferred over data splitting and cross validation for internal validation [37–41]. Model calibration was assessed using Hosmer-Lemeshow (HL) goodness-of-fit tests (null hypothesis of statistically significant difference between observed and predicted estimates), and residuals were evaluated to verify appropriate model fit [42].

### Sensitivity analyses

We conducted a sensitivity analysis to evaluate model performance using only study participants from resource-limited settings. Given the implementation and policy implications and hypothesized biological association of ART duration and drug resistance, we tested multiple forms of the treatment time variable (Additional file 1: Table S1-S3). Models 4–6 evaluate therapy duration categorized as <7, 7–24, and >24 months; models 7–9 dichotomized duration (<7 vs ≥7 months). We compared these alternatives to the primary models using AIC.

## Results

### Study population

Among 1045 participants, 305 had at least one VL ≥1000 copies/ml after week 16; 15 participants were excluded despite having two sequential VL ≥1000 copies/ml because resistance results were unavailable at the time of confirmed elevated VL, for a final sample of 290. Age ranged from 19 to 65 years, and 53 % of persons were male (Table 1). Mean CD4 at enrollment was 156 cells/mm^3^ and median VL at enrollment was 115,383 copies/ml.

**Table 1:** Bivariable association of need for ART change and potential predictor characteristics

Predictor	Overall (*n* = 290) N (%)	Resistant (*n* = 75)^a^ N (%)	Not resistant or resuppressed (*n* = 215) N (%)	Unadjusted prevalence OR (95 % CI)	*p*-value
Age, years					0.09
≤ 30	82 (28.3)	27 (36.0)	55 (25.6)	1.64 (0.93–2.87)	
> 30	208 (71.7)	48 (64.0)	160 (74.4)	1.0	
Sex					0.3
Male	154 (53.1)	36 (48.0)	118 (54.9)	0.76 (0.45, 1.28)	
Female	136 (46.9)	39 (28.7)	97 (71.3)	1.0	
BMI, kg/m^2^					0.002
Normal/low (<24.9)	229 (79.0)	50 (21.8)	179 (78.2)	1.0	
High (>25.0)	31 (21.0)	25 (41.0)	36 (59.0)	2.48 (1.37–4.52)	
CD4 at screening, cells/mm^3^					0.12
≤ 100	84 (71.0)	27 (36.0)	57 (26.5)	1.56 (0.89, 2.73)	
> 100	206 (29.0)	48 (23.3)	158 (76.7)	1.0	
Treatment initiation VL, copies/ml					0.001
≤ 100,000	135 (46.6)	23 (17.0)	112 (83.0)	1.0	
> 100,000	155 (53.4)	52 (33.5)	103 (66.5)	2.46 (1.41, 4.30)	
AIDS history					0.55
Yes	26 (9.0)	8 (30.8)	18 (69.2)	1.31 (0.54–3.14)	
No	264 (91.0)	67 (25.4)	197 (74.6)	1.0	
History of ART exposure					0.02
Yes	4 (1.4)	3 (75.0)	1 (25.0)	8.92 (0.91–87.1)	
No	286 (98.6)	72 (25.2)	214 (74.8)	1.0	
History of TB					0.14
Yes	60 (20.7)	11 (18.3)	49 (81.7)	1.0	
No	230 (79.3)	64 (27.8)	166 (72.2)	1.72 (0.84–3.51)	
Reported symptoms					0.22
Yes	37 (71.2)	11 (29.7)	26 (70.3)	2.75 (0.53–14.3)	
No	15 (28.9)	2 (13.3)	13 (86.7)	1.0	
Imperfect adherence					0.11
Yes	67 (25.6)	22 (32.8)	45 (67.2)	1.63 (0.89, 3.00)	
No	195 (74.4)	45 (23.1)	150 (76.9)	1.0	
Pill count, % taken					0.29
< 80 %	11 (22.4)	6 (54.5)	5 (45.5)	2.06 (0.53, 8.00)	
≥ 80 %	38 (77.6)	14 (36.8)	24 (63.2)	1.0	
Regimen frequency					0.84
Once daily (FTC/TDF/EFV QHS)	144 (49.7)	38 (26.4)	106 (73.6)	1.06 (0.62, 1.79)	
Twice daily (3TC/ZDV BID + EFV QHS)	146 (50.3)	37 (25.3)	109 (74.7)	1.0	
Time on therapy, months^b^					<0.001
< 7	102 (35.2)	42 (41.2)	60 (58.8)	5.1 (2.6–9.8)	
7–12	56 (19.3)	17 (30.4)	39 (69.6)	3.2 (1.5–6.8)	
> 12	132 (45.5)	16 (12.1)	116 (87.9)	1.0	
VL^c,^ copies/ml					<0.001
≤ 10,000	175 (60.4)	25 (14.3)	150 (85.7)	1.0	
10,001–100,000	70 (24.1)	34 (48.6)	36 (51.4)	5.7 (3.0–10.7)	
> 100,000	45 (15.5)	16 (35.6)	29 (64.4)	3.3 (1.6–6.9)	
CD4 at failure, cells/mm^3^					0.18
≤ 200	77 (27.6)	24 (31.2)	53 (68.8)	1.49 (0.83–2.7)	
> 200	202 (72.4)	47 (23.3)	155 (76.7)	1.0	
Any change in therapy during study					0.28
Yes	42 (14.5)	8 (19.1)	34 (80.1)	0.64 (0.38–1.4)	
No	248 (85.5)	67 (27.0)	181 (73.0)	1.0	

^a^Resistance indicates identified NRTI or NNRTI resistance mutations detected on stored specimens at time of first elevated (>1000 copies/ml) viral load

^b^Therapy duration defined by days, <7 months is <213; 7–12 months is 212–395, >12 months is >395 days

^c^Viral load at time of first VL ≥1000 copies/ml

*3TC* lamivudine, *ART* antiretroviral therapy, *BID* twice daily, *BMI* body-mass index, *CI* confidence interval, *EFV* efavirenz, *FTC* emtricitabine, *NNRTI* non-nucleoside reverse transcriptase inhibitor, *NRTI* nucleoside reverse transcriptase inhibitor, *OR* odds ratio, *QHS* nightly, *TB* tuberculosis, *TDF* tenofovir, *VL* viral load, *ZDV* zidovudine

### Bivariable analyses

Among the 290 with at least one VL ≥1000 copies/ml, 53 % (148/290) resuppressed at the next visit. Among the remaining 142 who did not resuppress, 75 had resistance mutations (either transmitted or acquired) to NRTI or NNRTI drugs. Thus, overall, NRTI or NNRTI resistance was detected in 25.9 % (95 % CI 20.8 %, 30.9 %) of participants with at least one VL ≥1000 copies/ml. Participants with a higher VL at ART initiation (>100,000 copies/ml) (OR = 2.5, 95 % CI 1.4, 4.3) were more likely to need ART change than participants with a lower VL at ART initiation (Table 1). At time of VL elevation, VL >100,000 copies/ml (OR = 3.3, 95 % CI 1.6, 6.9) or 10,000–100,000 copies/ml (OR = 5.7, 95 % CI 3.0, 10.7) also were associated with increased likelihood of needing ART change, compared to participants with VL <10,000 copies/ml. Participants who were on therapy <7 months (OR = 5.1, 95 % CI 2.6, 9.8), or 7–12 months (OR = 3.2, 95 % CI 1.5, 6.8) were more likely to need ART change than participants on therapy >12 months. Participants whose BMI > 25.0 kg/m^2^ at ART initiation were more likely to need ART change at time of first VL ≥1000 copies/ml than participants with BMI ≤25.0 kg/m^2^ (OR = 2.5, 95 % CI 1.4, 4.5).

### Multivariable analyses

#### Model 1 - Including treatment initiation VL and CD4

The full model included ten predictor variables (AUROC = 0.842) and showed acceptable HL test, failing to reject the null hypothesis (*p* = 0.70). Our final model contained five predictor variables: age <30, BMI > 25.0, treatment initiation VL ≤100,000 copies/ml, time on treatment, and VL at time of first VL ≥1000 (Table 2). The AUROC was 0.8165 for the reduced model, which showed acceptable calibration, (HL p = 0.12)

**Table 2:** Adjusted odds ratios and risk scores of need for ART change

Predictor	Model 1 (with baseline VL) (*n* = 290), AUROC = 0.8165				Model 2 (without baseline VL) (*n* = 290), AUROC = 0.7981				Model 3 (without baseline VL or CD4) (*n* = 260), AUROC = 0.7937			
	Full model OR (95 % CI)	Reduced OR (95 % CI)	β^b^	Predictor score^a^	Full model OR (95 % CI)	Reduced model OR (95 % CI)	β^c^	Predictor score^a^	Full model OR (95 % CI)	OR (95 % CI)	β^d^	Predictor score^a^
Age, years												
≤ 30	2.2 (1.0–4.6)	2.1 (1.0–4.1)	0.72	1	1.8 (0.9–3.7)	1.8 (0.9–3.5)	0.59	1	1.6 (0.8–3.2)	1.7 (0.9–3.4)	0.53	1
> 30	1.0	1.0		0	1.0	1.0		0	1.0	1.0		0
Sex												
Male	0.7 (0.4–1.4)	- -	-	-	0.7 (0.3–1.3)	- -	-	-	0.7 (0.4–1.4)	- -	-	-
Female	1.0	- -	-	-	1.0	- -	-	-	1.0	- -	-	-
BMI, kg/m^2^												
Normal/low (<24.9)	1.0	1.0		0	1.0	1.0		0	1.0	1.0		0
High (>25.0)	2.8 (1.2–6.4)	3.7 (1.8–7.8)	1.31	2	2.5 (1.1–5.6)	3.2 (1.6–6.6)	1.18	2	2.3 (1.1–4.1)	2.7 (1.2–5.7)	0.98	2
Treatment initiation VL, copies/ml												
≤ 100,000	1.0	1.0		0	- -	- -	-	-	- -	- -	-	-
> 100,000	3.2 (1.5–7.1)	3.6 (1.8–7.0)	1.27	2	- -	- -	-	-	- -	- -	-	-
Time on therapy, months												
< 7	4.2 (1.9–9.2)	4.2 (2.0–8.6)	1.43	3	3.9 (1.8–8.3)	4.3 (2.1–8.7)	1.45	3	3.6 (1.7–7.6)	3.7 (1.8–7.8)	1.32	3
7–12	2.0 (0.8–5.1)	2.9 (1.2–6.9)	1.07	2	1.9 (0.8–4.8)	3.1 (1.3–7.2)	1.13	2	1.9 (0.8–4.8)	2.1 (0.9–5.2)	0.76	2
> 12	1.0	1.0		0	1.0	1.0		0		1.0		0
VL^e^, copies/ml												
≤ 10,000	1.0	1.0		0	1.0	1.0		0	1.0	1.0		0
10,001–100,000	7.3 (3.4–15.9)	6.3 (3.1–13.0)	1.85	4	7.4 (3.4–15.8)	6.3 (3.1–12.8)	1.85	4	6.5 (3.1–13.5)	6.5 (3.1–13.3)	1.87	4
> 100,000	2.8 (1.1–7.2)	2.7 (1.2–6.1)	0.99	2	2.7 (1.1–6.8)	3.1 (1.4–7.0)	1.15	2	2.7 (1.1–6.6)	3.0 (1.2–7.2)	1.10	2
CD4 at screening, cells/mm^3^												
≤ 100	1.8 (0.9–3.9)	- -	-	-	2.6 (1.3–5.3)	2.2 (1.2–4.3)	0.81	2	- -	- -	-	-
> 100	1.0	- -	-	-	1.0	1.0		0	- -	- -	-	-
History of TB												
Yes	1.0	- -	-	-	1.0	- -	-	-	1.0	- -	-	-
No	1.8 (0.7–4.5)	- -	-	-	1.3 (0.6–3.2)	- -	-	-	1.3 (0.6–3.2)	- -	-	-
Treatment changed while on study												
Yes	0.4 (0.1–1.3)	- -	-	-	0.4 (0.1–1.2)	- -	-	-	0.4 (0.1–1.3)	- -	-	-
No	1.0	- -	-	-	1.0	- -	-	-	1.0	- -	-	-
Ever missed meds												
Yes	1.8 (0.9–3.7)	- -	-	-	2.1 (1.0–4.1)	- -	-	-	2.1 (1.0–4.1)	1.8 (1.0–3.6)	0.61	1
No	1.0	- -	-	-	1.0	- -	-	-		1.0		0

^a^weighted; ^b^constant = −3.94; ^c^constant = −3.42; ^d^constant = −3.11

^e^Viral load at time of first VL ≥1000 copies/ml

*CI* confidence interval, *β* beta regression coefficient, *BMI* body mass index, *NNRTI* non-nucleoside reverse transcriptase inhibitor, *NRTI* nucleoside reverse transcriptase inhibitor, *OR* odds ratio, *AUROC* area under receiver operating characteristic curve, *VL* viral load

#### Model 2 - Excluding treatment initiation VL

The full model included nine predictor variables (AUROC = 0.819) and showed acceptable calibration (HL failed to reject null, *p* = 0.84). The reduced model contained six predictor variables (AUROC = 0.807). To meet the predefined criterion of a five-variable model, we eliminated the variable with the lowest OR (self-reported adherence). Our final model contained: age <30, screening CD4 < 100 cells/mm^3^, BMI >25.0, time on treatment, and VL at time of first VL ≥1000 (AUROC = 0.7981) (Table 2). The reduced model showed acceptable calibration (HL p = 0.84).

#### Model 3 - Excluding treatment initiation VL and CD4

The full model included eight predictor variables (AUROC = 0.801) and showed acceptable calibration (HL p = 0.37). The final model contained: age <30, self-reported missed medications, BMI > 25.0, time on treatment, and VL at time of first VL ≥1000 (AUROC = 0.7937) (Table 2). The reduced model showed acceptable calibration (HL, p = 0.10).

**Fig. 2:**
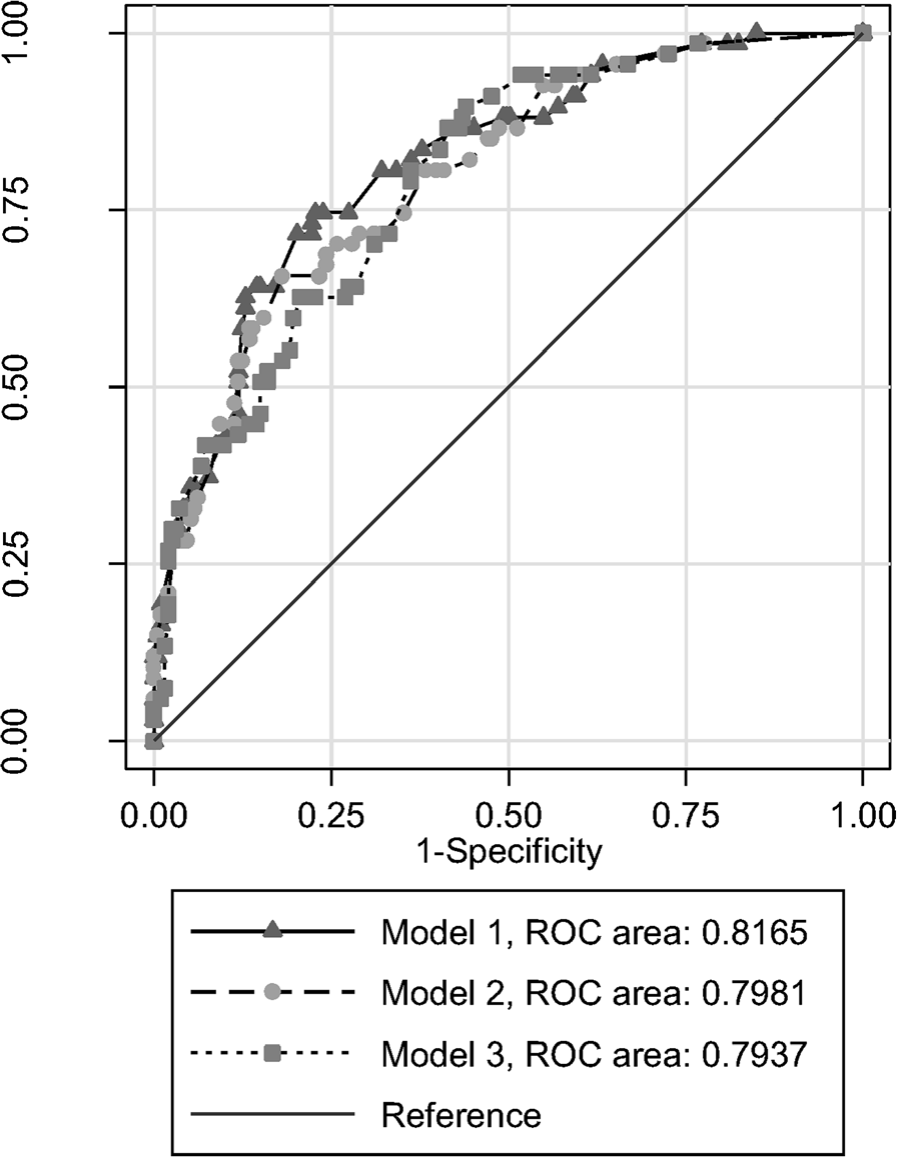
Receiver operating characteristic (ROC) curves for Models 1–3. The area under an ROC curve is a measure of model performance. Specifically, the area measures discrimination – in this case the ability of the predictive model to correctly classify persons with and without resistance. Model 1, in which we assumed that viral load and CD4 cell counts from time of treatment initiation were available, performed the best, and had an area under the ROC curve of 0.8165. In Model 2, when viral load from treatment initiation was excluded as an eligible predictor, performed slightly less well (area under ROC curve of 0.7981). Finally, in Model 3, we assumed that neither viral load nor CD4 cell counts from time of treatment initiation were available. This model performed the poorest of all three models evaluated, with an area under the ROC curve of 0.794 – although this difference was not statistically significant and may not be clinically meaningful

Reduced Model 1 performed slightly better than reduced Model 2, but the difference was not significant (*p* = 0.23). Reduced Model 3 performed slightly worse again, but compared to reduced Model 1, the difference was not statistically significant (*p* = 0.22) (Fig. 2). Bootstrapping demonstrated consistent performance for all models over 1000 replications. Further model diagnostics demonstrated normally distributed residuals; graphs of predicted probabilities against residuals suggested random distribution.

### Risk scores

The weighted risk scores ranged from 0–12 for Models 1 and 2, and 0–11 for Model 3 (Table 2). The maximum attained score by any individual in the tested population was 11 for each model. The predictive power of the model was retained when predicted probabilities were transformed to risk scores (AUROC for Model 1 = 0.813 (*p* = 0.69), Model 2 = 0.797 (*p* = 0.91), and Model 3 = 0.802 (*p* = 0.57)). A risk score cutoff of ≥9 met predefined specificity threshold (>95.0 %) (Table 3).

**Table 3:** Performance of models and derived risk scores

Predictor	Model with baseline VL (*n* = 290)			Model without baseline VL (*n* = 290)			Model without baseline VL or CD4 (*n* = 260)		
	Cutoff	Sensitivity	Specificity	Cutoff	Sensitivity	Specificity	Cutoff	Sensitivity	Specificity
Unrestricted (RLS & non-RLS)									
Model^a^	0.657	22.7 %	98.1 %	0.640	22.7 %	97.2 %	0.741	13.4 %	98.4 %
Weighted risk score	≥9	28.0 %	96.7 %	≥9	16.0 %	98.6 %	≥9	14.7 %	98.1 %
Restricted (RLS only)									
Model^a^	0.653	28.0 %	97.2 %	0.697	18.7 %	98.1 %	0.691	14.9 %	98.4 %
Weighted risk score	≥9	26.0 %	97.4 %	≥9	10.0 %	99.5 %	≥9	14.0 %	99.5 %

^a^Cutoff values for the models are thresholds derived by summing the beta coefficients and converting to a probability RLS, resource-limited setting; VL, viral load

**Fig. 3:**
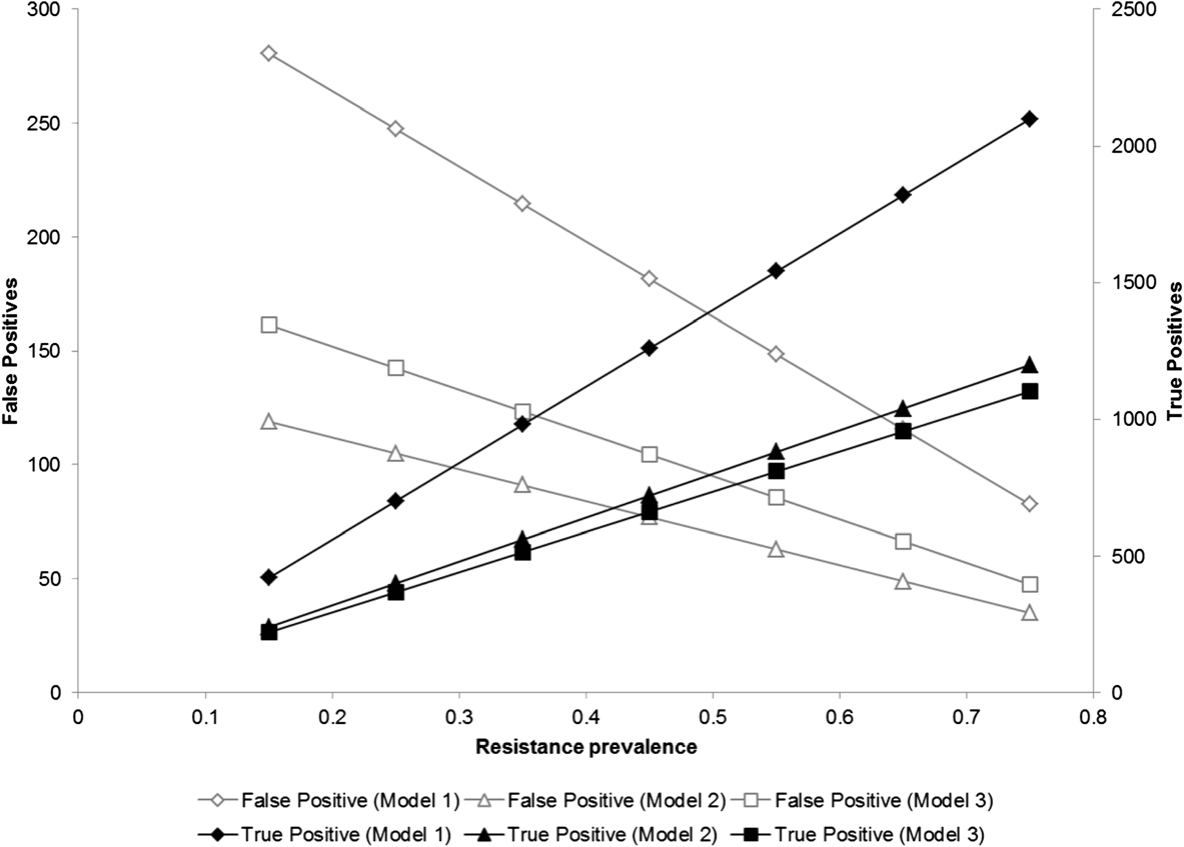
Number of false positive and true positive results in hypothetical cohort of 10,000 ART patients with elevated viral load at varied resistance prevalence estimates. Using the sensitivities and specificities for each risk score at the defined threshold, we generated the number of false positives and true positives that would be expected among a 10,000-person cohort of patients with an initially elevated viral load. We evaluated these outcomes at varying levels of ART resistance. As the prevalence of resistance increases, the positive predictive value of the risk scores also improves

We estimated the number of patients who would be immediately switched to second-line therapy in a hypothetical population of 10,000 ART patients receiving VL monitoring. Given the proportion of patients who did not resuppress and who harbored drug resistance (~25 % of entire study population), Model 1 risk score would accurately identify 700 persons who needed ART change (true positives) and incorrectly classify 248 persons as needing ART change when they did not (false positives) (Fig. 3). At this same drug resistance prevalence, Model 2 would correctly switch 400 persons in need of ART change and have 105 false positives. Model 3 would correctly switch 368 persons in need of ART change, with 143 false positives. However, as the underlying drug resistance prevalence increases, so too does the number of true positives as well as the ratio of true positive:false positive. For example, with a prevalence of 55 % in a population of 10,000 ART patients with a VL ≥1000 copies/ml, Model 1 would correctly identify 1540 patients as needing ART change with only 149 false positives.

### Sensitivity analyses

Model performance was comparable when the study population was restricted to persons from resource-limited settings: AUROC =0.823 (Model 1), 0.812 (Model 2), and 0.804 (Model 3). Using the same risk score cutoff as in the unrestricted model (≥9), the sensitivity for the three models ranged from 10.0–26.0 %; specificity ranged from 97.4–99.5 % (Table 3).

## Discussion

Current WHO guidelines recommend confirmatory testing for ART patients with high VL (≥1000 copies/ml). A subset of patients will be resistant at the time of initial elevated VL; for these persons, requiring confirmatory testing unnecessarily delays switch to second-line therapy. We developed a risk score using only parameters that are likely to be available to providers in resource-limited settings that successfully identifies person with persistently elevated VL or are resistant and need immediate ART change. The risk score performed well, >98 % specific in most model iterations. Although increased specificity comes at the cost of the lower sensitivity (~15–30 %), this is less concerning as these “misses” will undergo confirmatory testing as is the standard-of-care for virological failure [1]. Rapidly switching patients with resistance to more efficacious second-line therapy could reduce transmission of resistant viral strains and transmission overall, and could prevent further declines in CD4 cell counts, resulting in meaningful public health benefits.

Utilizing this risk score may also reduce costs by avoiding unnecessary confirmatory VL tests. Alternative cost-saving strategies for virological monitoring include pooling specimens and targeting VL tests based on clinical or immunological criteria [1, 43–47]. Despite potential cost-savings, pooling requires additional laboratory support for linkage and deconstruction of positive pools. Applying a conservative estimate of treatment failure (16.0 % at 12 months) would translate to >1,000,000 ART patients having an elevated VL in sub-Saharan Africa alone [20]. Even a modest reduction in confirmatory test volume resulting from implementation of our algorithm could substantially reduce expenditures and patients’ clinical deterioration.

Our risk score balances predictive ability and practicality. Notably, given our goal of point-of-care application, we considered only predictors that were likely available in ART clinics within resource-limited settings. We also sacrificed some precision for ease-of-use by collapsing continuous variables into discrete categories and limiting the number of included variables.

Maximizing specificity was essential to decrease false positives. We selected 95.0 % as the lower threshold for specificity, though selected risk score thresholds had higher specificities (96.7–98.6 %). Even at specificities >98 %, prematurely switching a patient to second-line therapy (false positive) still occurs and has significant person- and system-wide consequences. For patients, false positive misclassification results in lost potential life years from remaining on first-line therapy. These are patients who, with improved adherence, may resuppress. For the healthcare system, premature second-line switching results in increased drug costs– as much as 6–10 times the cost of first-line therapy [26, 48]. Conversely, missing patients who are resistant is also associated with substantial health consequences and healthcare system costs, including accumulation and potential transmission of resistant viral strains. The current study used the WHO-accepted threshold of <1000 copies/ml for defining resuppression, and assumed that persons who resuppressed were not harboring clinically significant resistance mutations. However, resistance may still be present at low-level viremia (<1000 copies/ml) [49–53], and may be associated with subsequent virological failure [54], suggesting that policies for treatment change thresholds, and thus the proposed algorithm, may misclassify the need for ART switch in the presence of resistance at lower viral loads. Modeling the consequences of delayed second-line initiation versus premature switching may help elucidate the trade-offs inherent to these thresholds. Importantly, trade-offs may vary by population: for example, providers may be more willing to “risk” false positive results in HIV-infected pregnant women given that viral suppression at time of delivery prevents vertical transmission. Acceptable true positive:false positive ratios may also differ depending on anticipated time-to-referral, as the patient and public health benefits of immediate switching may be greater in settings with extensive delays in second-line initiation [18, 19].

These data came from a controlled clinical trial, and enrolled participants may not be representative of larger ART populations. Viral suppression was similar to other cohorts with nearly 30 % of participants having a VL ≥1000 copies/ml after ≥16 weeks on ART [20]. Participants received frequent virological monitoring (every 8 weeks) in the study, which is unlikely in the intended settings for this risk score. The risk score used >12 months as the referent category, however, sensitivity analyses with alternative categorization of therapy duration did not change model performance (Additional file 1). Furthermore, all patients included in this analysis were initiated on efavirenz-based first-line regimens – resistance patterns and predictors may not apply to non-efavirenz-based regimens depending on different barriers to resistance mutations. Importantly, participants were recruited largely from resource-limited settings and the risk score performed well in this subgroup. Furthermore, PEARL’s broad inclusion criteria improves generalizability. Study-driven CD4 cell count eligibility were consistent with WHO guidelines (<300 cells/mm^3^), but these guidelines have since changed, expanding ART eligibility to HIV-infected persons earlier in the course of disease (<500 cells/mm^3^) [1]. If CD4 is included (Model 2), having more patients with high CD4 at treatment initiation could mean that fewer patients reach the switch score threshold, potentially dampening the efficiency gains of the algorithm.

Among patients with ≥1 elevated VL, resistance rates were lower than observed in sub-Saharan African cohorts (as high as 70 %) [2, 11, 55–57], however, this may result from more frequent VL monitoring in the study, which increases the likelihood of detecting transiently elevated VL. A higher prevalence of resistance would favor use of the risk score, increasing the score’s positive predictive value. Assuming 55 % resistance among patients with an elevated VL, we demonstrated that in a hypothetical cohort of 10,000 ART patients, >1500 would be appropriately classified as resistant and switched immediately, with only 150 false positives.

## Conclusions

To our knowledge, this risk score is the first to identify the need for immediate ART change among persons with a single elevated VL. We successfully identified predictors that reliably distinguished between persons who do and do not need immediate ART change from first-line regimens. Our risk score is sensitive to realities in resource-limited settings: we used a limited number of readily-available categorical variables and minimized false positive results. This model is a promising opportunity to quickly transition patients with probable resistance to more effective regimens – improving ART morbidity and mortality outcomes. Using this risk score may reduce transmission of resistant viral strains and save healthcare systems scarce resources by reducing personnel and equipment costs incurred with unnecessary confirmatory VL testing. These potential benefits should be assessed and externally validated prospectively by evaluating the effect of the risk score on health outcomes and resource utilization, taking into account the trade-offs associated with misclassifying even a small subset of patients as needing ART change when they do not [31].

## Abbreviations

ACTG, AIDS Clinical Trials Group; AIC, Akaike’s information criteria; ART, antiretroviral therapy; AUROC, area under the receiver operating characteristic; BMI, body mass index; CI, confidence intervals; HL, Hosmer-Lemeshow; LR, likelihood ratio; NNRTI, non-nucleoside reverse transcriptase inhibitor; NRTI, nucleoside reverse transcriptase inhibitor; OR, odds ratios; PEARLS, Prospective Evaluation of Antiretrovirals in Resource-Limited Settings; PI, protease inhibitors; VL, viral load; WHO, World Health Organization

## Additional file

Additional file 1: Supplementary Tables, S1–S3. (DOC 185 kb)
