# Supplementary material for: Predicting resistance as indicator for need to switch from first-line antiretroviral therapy among patients with elevated viral loads: development of a risk score algorithm
Source: BMC Infect Dis. 2016 Jun 13;16:280. doi: 10.1186/s12879-016-1611-2 (PMC4906700; doi:10.1186/s12879-016-1611-2)
Supplement: Additional file 1: — Supplementary Tables, S1–S3. (DOC 185 kb) [file 12879_2016_1611_MOESM1_ESM.doc]

Additional file 1

| **Table S1: Sensitivity Analysis Adjusted Odds Ratios and Risk Scores of NRTI/NNRTI resistance (Time on therapy, <7mo, 7-24mo, >24mo)** | | | | | | | | | | | | | | | | | | | | | | |
| --- | --- | --- | --- | --- | --- | --- | --- | --- | --- | --- | --- | --- | --- | --- | --- | --- | --- | --- | --- | --- | --- | --- |
| Predictor | | | Model 4 (with baseline VL) (n=290), AUROC=0.815 | | | | | |  | Model 5 (without baseline VL) (n=290), AUROC=0.794 | | | | | |  | Model 6 (without baseline VL or CD4) (n=260), AUROC =0.794 | | | | | |
|  | | | Full model  OR (95% CI) | | Reduced  OR (95% CI) | | β^2^ | Predictor score^1^ |  | Full model  OR (95% CI) | | Reduced model  OR (95% CI) | | β^3^ | Predictor score^1^ |  | Full model  OR (95% CI) | | OR (95% CI) | | β^4^ | Predictor score^1^ |
| Age, years | | |  |  |  |  |  |  |  |  |  |  |  |  |  |  |  |  |  |  |  |  |
|  | | ≤30 | 2.1 | (1.0-4.5) | 2.0 | (1.0-4.0) | 0.71 | 1 |  | 1.8 | (0.9-3.6) | 1.8 | (0.9-3.4) | 0.57 | 1 |  | 1.5 | (0.8-3.1) | 1.7 | (0.8-3.4) | 0.52 | 1 |
|  | | >30 | 1.0 |  | 1.0 |  |  | 0 |  | 1.0 |  | 1.0 |  |  | 0 |  | 1.0 |  | 1.0 |  |  | 0 |
| Sex | | |  |  |  |  |  |  |  |  |  |  |  |  |  |  |  |  |  |  |  |  |
|  | Male | | 0.7 | (0.3-1.4) | - | - | - | - |  | 0.7 | (0.3-1.3) | - | - | - | - |  | 0.7 | (0.4-1.4) | - | - | - | - |
|  | Female | | 1.0 |  | - | - | - | - |  | 1.0 |  | - | - | - | - |  | 1.0 |  | - | - | - | - |
| BMI, kg/m^2^ | | |  |  |  |  |  |  |  |  |  |  |  |  |  |  |  |  |  |  |  |  |
|  | | Normal/low (<24.9) | 1.0 |  | 1.0 |  |  | 0 |  | 1.0 |  | 1.0 |  |  | 0 |  | 1.0 |  | 1.0 |  |  | 0 |
|  | | High (>25.0) | 2.9 | (1.3-6.5) | 3.9 | (1.9-8.1) | 1.35 | 3 |  | 2.5 | (1.1-5.6) | 3.3 | (1.6-6.7) | 1.20 | 2 |  | 2.3 | (1.1-5.1) | 2.7 | (1.3-5.7) | 0.99 | 2 |
| Baseline VL, copies/ml | | |  |  |  |  |  |  |  |  |  |  |  |  |  |  |  |  |  |  |  |  |
|  | | ≤100,000 | 1.0 |  | 1.0 |  |  | 0 |  | - | - | - | - | - | - |  | - | - | - | - | - | - |
|  | | >100,000 | 3.2 | (1.5-7.1) | 3.7 | (1.9-7.2) | 1.30 | 3 |  | - | - | - | - | - | - |  | - | - | - | - | - | - |
| Time on therapy, months | | |  |  |  |  |  |  |  |  |  |  |  |  |  |  |  |  |  |  |  |  |
|  | | <7 | 5.3 | (1.8-15.6) | 5.6 | (2.1-15.1) | 1.72 | 3 |  | 4.9 | (1.7-14.0) | 5.7 | (2.1-15.1) | 1.73 | 3 |  | 4.4 | (1.6-12.3) | 4.7 | (1.7-13.0) | 1.55 | 3 |
|  | | 7-24 | 2.0 | (0.7-5.8) | 2.6 | (1.0-7.1) | 0.96 | 2 |  | 1.9 | (0.7-5.5) | 2.7 | (1.0-7.3) | 1.0 | 2 |  | 1.8 | (0.7-5.1) | 2.0 | (0.7-5.5) | 0.69 | 1 |
|  | | >24 | 1.0 |  | 1.0 |  |  | 0 |  | 1.0 |  | 1.0 |  |  | 0 |  | 1.0 |  | 1.0 |  |  | 0 |
| VL at failure, copies/ml | | |  |  |  |  |  |  |  |  |  |  |  |  |  |  |  |  |  |  |  |  |
|  | | ≤10,000 | 1.0 |  | 1.0 |  |  | 0 |  | 1.0 |  | 1.0 |  |  | 0 |  | 1.0 |  | 1.0 |  |  | 0 |
|  | | 10,001-100,000 | 7.1 | (3.3-15.3) | 6.0 | (3.0-12.3) | 1.80 | 4 |  | 7.1 | (3.4-15.2) | 5.9 | (3.0-11.9) | 1.78 | 4 |  | 6.3 | (3.0-13.1) | 6.3 | (3.1-12.9) | 1.84 | 4 |
|  | | >100,000 | 2.7 | (1.0-7.0) | 2.6 | (1.2-5.9) | 0.97 | 2 |  | 2.7 | (1.1-6.7) | 3.1 | (1.4-6.8) | 1.12 | 2 |  | 2.7 | (1.1-6.5) | 2.9 | (1.2-7.1) | 1.07 | 2 |
| CD4 at screening, cells/mm^3^ | | |  |  |  |  |  |  |  |  |  |  |  |  |  |  |  |  |  |  |  |  |
|  | | ≤100 | 1.9 | (0.9-3.9) | - | - | - | - |  | 2.7 | (1.3-5.4) | 2.3 | (1.2-4.3) | 0.82 | 2 |  | - | - | - | - | - | - |
|  | | >100 | 1.0 |  | - | - | - | - |  | 1.0 |  | 1.0 |  |  | 0 |  | - | - | - | - | - | - |
| History of TB | | |  |  |  |  |  |  |  |  |  |  |  |  |  |  |  |  |  |  |  |  |
|  | Yes | | 1.0 |  | - | - | - | - |  | 1.0 |  | - | - | - | - |  | 1.0 |  | - | - | - | - |
|  | No | | 1.8 | (0.7-4.6) | - | - | - | - |  | 1.4 | (0.6-3.3) | - | - | - | - |  | 1.4 | (0.6-3.2) | - | - | - | - |
| Treatment changed while on study | | |  |  |  |  |  |  |  |  |  |  |  |  |  |  |  |  |  |  |  |  |
|  | Yes | | 0.4 | (0.1-1.3) | - | - | - | - |  | 0.4 | (0.1-1.1) | - | - | - | - |  | 0.4 | (0.1-1.3) | - | - | - | - |
|  | No | | 1.0 |  | - | - | - | - |  | 1.0 |  | - | - | - | - |  | 1.0 |  | - | - | - | - |
| Ever missed meds | | |  |  |  |  |  |  |  |  |  |  |  |  |  |  |  |  |  |  |  |  |
|  | | Yes | 2.0 | (1.0-4.0) | - | - | - | - |  | 2.2 | (1.1-4.3) | - | - | - | - |  | 2.2 | (1.1-4.3) | 2.0 | (1.0-3.8) | 0.68 | 1 |
|  | | No | 1.0 |  | - | - | - | - |  | 1.0 |  | - | - | - | - |  | 1.0 |  | 1.0 |  |  | 0 |
| ^1^weighted; ^2^constant = -4.23; ^3^constant = -3.67; ^4^constant = -3.34  CI, confidence interval; β, regression coefficient; BMI, body mass index; NNRTI, non-nucleoside reverse transcriptase inhibitor; NRTI, nucleoside reverse transcriptase inhibitor; OR, odds ratio; AUROC, area under receiver operating characteristic curve; VL, viral load | | | | | | | | | | | | | | | | | | | | | | |

| **Table S2: Sensitivity Analysis Adjusted Odds Ratios and Risk Scores of NRTI/NNRTI resistance (Time on therapy, <7mo, ≥7mo)** | | | | | | | | | | | | | | | | | | | | | | |
| --- | --- | --- | --- | --- | --- | --- | --- | --- | --- | --- | --- | --- | --- | --- | --- | --- | --- | --- | --- | --- | --- | --- |
| Predictor | | | Model 7 (with baseline VL) (n=290), AUROC=0.802 | | | | | |  | Model 8 (without baseline VL) (n=290), AUROC=0.786 | | | | | |  | Model 9 (without baseline VL or CD4) (n=260), AUROC =0.787 | | | | | |
|  | | | Full model  OR (95% CI) | | Reduced  OR (95% CI) | | β^2^ | Predictor score^1^ |  | Full model  OR (95% CI) | | Reduced model  OR (95% CI) | | β^3^ | Predictor score^1^ |  | Full model  OR (95% CI) | | OR (95% CI) | | β^4^ | Predictor score^1^ |
| Age, years | | |  |  |  |  |  |  |  |  |  |  |  |  |  |  |  |  |  |  |  |  |
|  | | ≤30 | 2.2 | (1.0-4.6) | 2.1 | (1.1-4.1) | 0.74 | 1 |  | 1.8 | (0.9-3.7) | 1.9 | (1.0-3.6) | 0.62 | 1 |  | 1.6 | (0.8-3.2) | 1.7 | (0.9-3.5) | 0.55 | 1 |
|  | | >30 | 1.0 |  | 1.0 |  |  | 0 |  | 1.0 |  | 1.0 |  |  | 0 |  | 1.0 |  | 1.0 |  |  | 0 |
| Sex | | |  |  |  |  |  |  |  |  |  |  |  |  |  |  |  |  |  |  |  |  |
|  | Male | | 0.7 | (0.3-1.4) | - | - | - | - |  | 0.7 | (0.3-1.3) | - | - | - | - |  | 0.7 | (0.4-1.4) | - | - | - | - |
|  | Female | | 1.0 |  | - | - | - | - |  | 1.0 |  | - | - | - | - |  | 1.0 |  | - | - | - | - |
| BMI, kg/m^2^ | | |  |  |  |  |  |  |  |  |  |  |  |  |  |  |  |  |  |  |  |  |
|  | | Normal/low (<24.9) | 1.0 |  | 1.0 |  |  | 0 |  | 1.0 |  | 1.0 |  |  | 0 |  | 1.0 |  | 1.0 |  |  | 0 |
|  | | High (>25.0) | 2.8 | (1.2-6.2) | 3.8 | (1.8-7.8) | 1.33 | 2 |  | 2.5 | (1.1-5.5) | 3.3 | (1.7-6.7) | 1.20 | 2 |  | 2.3 | (1.1-5.1) | 2.7 | (1.3-5.7) | 0.98 | 2 |
| Baseline VL, copies/ml | | |  |  |  |  |  |  |  |  |  |  |  |  |  |  |  |  |  |  |  |  |
|  | | ≤100,000 | 1.0 |  | 1.0 |  |  | 0 |  | - | - | - | - | - | - |  | - | - | - | - | - | - |
|  | | >100,000 | 3.2 | (1.5-6.9) | 3.6 | (1.8-7.1) | 1.29 | 3 |  | - | - | - | - | - | - |  | - | - | - | - | - | - |
| Time on therapy, months | | |  |  |  |  |  |  |  |  |  |  |  |  |  |  |  |  |  |  |  |  |
|  | | <7 | 3.2 | (1.6-6.4) | 2.8 | (1.5-5.2) | 1.03 | 2 |  | 3.0 | (1.6-5.9) | 2.7 | (1.5-5.0) | 1.01 | 2 |  | 2.8 | (1.5-5.4) | 2.8 | (1.5-5.4) | 1.04 | 2 |
|  | | ≥7 | 1.0 |  | 1.0 |  |  | 0 |  | 1.0 |  | 1.0 |  |  | 0 |  | 1.0 |  | 1.0 |  |  | 0 |
| VL at failure, copies/ml | | |  |  |  |  |  |  |  |  |  |  |  |  |  |  |  |  |  |  |  |  |
|  | | ≤10,000 | 1.0 |  | 1.0 |  |  | 0 |  | 1.0 |  | 1.0 |  |  | 0 |  | 1.0 |  | 1.0 |  |  | 0 |
|  | | 10,001-100,000 | 7.3 | (3.4-15.6) | 6.3 | (3.1-12.7) | 1.84 | 4 |  | 7.3 | (3.5-15.6) | 6.2 | (3.1-12.3) | 1.82 | 4 |  | 6.5 | (3.1-13.5) | 6.4 | (3.1-13.2) | 1.86 | 4 |
|  | | >100,000 | 2.7 | (1.0-6.9) | 2.7 | (1.2-5.9) | 0.98 | 2 |  | 2.6 | (1.1-6.5) | 3.0 | (1.4-6.6) | 1.10 | 2 |  | 2.6 | (1.1-6.3) | 2.8 | (1.2-6.8) | 1.04 | 2 |
| CD4 at screening, cells/mm^3^ | | |  |  |  |  |  |  |  |  |  |  |  |  |  |  |  |  |  |  |  |  |
|  | | ≤100 | 1.9 | (0.9-3.9) | - | - | - | - |  | 2.7 | (1.3-5.4) | 2.2 | (1.2-4.2) | 0.79 | 2 |  | - | - | - | - | - | - |
|  | | >100 | 1.0 |  | - | - | - | - |  | 1.0 |  | 1.0 |  |  | 0 |  | - | - | - | - | - | - |
| History of TB | | |  |  |  |  |  |  |  |  |  |  |  |  |  |  |  |  |  |  |  |  |
|  | Yes | | 1.0 |  | - | - | - | - |  | 1.0 |  | - | - | - | - |  | 1.0 |  | - | - | - | - |
|  | No | | 1.9 | (0.8-4.8) | - | - | - | - |  | 1.5 | (0.6-3.4) | - | - | - | - |  | 1.4 | (0.6-3.3) | - | - | - | - |
| Treatment changed while on study | | |  |  |  |  |  |  |  |  |  |  |  |  |  |  |  |  |  |  |  |  |
|  | Yes | | 0.4 | (0.1-1.2) | - | - | - | - |  | 0.4 | (0.1-1.1) | - | - | - | - |  | 0.4 | (0.1-1.2) | - | - | - | - |
|  | No | | 1.0 |  | - | - | - | - |  | 1.0 |  | - | - | - | - |  | 1.0 |  | - | - | - | - |
| Ever missed meds | | |  |  |  |  |  |  |  |  |  |  |  |  |  |  |  |  |  |  |  |  |
|  | | Yes | 2.0 | (1.0-3.9) | - | - | - | - |  | 2.2 | (1.1-4.3) | - | - | - | - |  | 2.2 | (1.1-4.3) | 2.0 | (1.0-3.8) | 0.68 | 1 |
|  | | No | 1.0 |  | - | - | - | - |  | 1.0 |  | - | - | - | - |  | 1.0 |  | 1.0 |  |  | 0 |
| ^1^weighted; ^2^constant = -3.56; ^3^constant = -2.97; ^4^constant = -2.86  CI, confidence interval; β, regression coefficient; BMI, body mass index; NNRTI, non-nucleoside reverse transcriptase inhibitor; NRTI, nucleoside reverse transcriptase inhibitor; OR, odds ratio; ROC, receiver operating characteristic; VL, viral load | | | | | | | | | | | | | | | | | | | | | | |

| **Table S3: Sensitivity analysis performance of resistance models and derived risk scores** | | | | | | | | | | | | |
| --- | --- | --- | --- | --- | --- | --- | --- | --- | --- | --- | --- | --- |
| Predictor | | Model with baseline VL (n=290) | | |  | Model without baseline VL (n=290) | | |  | Model without baseline VL or  CD4 (n=260) | | |
|  |  | Cutoff | Sensitivity | Specificity |  | Cutoff | Sensitivity | Specificity |  | Cutoff | Sensitivity | Specificity |
| <7mo, 7-24mo, >24mo | |  |  |  |  |  |  |  |  |  |  |  |
|  | Model* | 0.656 (4) | 17.3% | 97.7% |  | 0.614 (5) | 22.7% | 97.2% |  | 0.635 (6) | 23.9% | 97.9% |
|  | Weighted risk score | ≥10^1^ | 34.7% | 95.3% |  | ≥9^2^ | 16.0% | 98.1% |  | ≥9^3^ | 14.7% | 98.1% |
| <7mo vs ≥7mo | |  |  |  |  |  |  |  |  |  |  |  |
|  | Model* | 0.656 (7) | 17.3% | 97.7% |  | 0.634 (8) | 18.7% | 97.2% |  | 0.646 (9) | 23.9% | 97.9% |
|  | Weighted risk score | ≥9^4^ | 26.7% | 95.8% |  | ≥8^5^ | 13.3% | 98.1% |  | ≥9^6^ | 24.0% | 97.2% |
| *Cutoff values for the models are thresholds derived by summing the beta coefficients and converting to a probability  ^1^Range of scores for this model was 0-14; ^2^Range of scores for this model was 0-12; ^3^Range of scores for this model was 0-11; ^4^Range of scores for this model was 0-12; ^5^ Range of scores for this model was 0-11; ^6^Range of scores for this model was 0-10  Mo, months; RLS, resource-limited setting; VL, viral load | | | | | | | | | | | | |
